# Head-to-head study of diagnostic accuracy of plasma and cerebrospinal fluid *p*-tau217 versus *p*-tau181 and *p*-tau231 in a memory clinic cohort

**DOI:** 10.1007/s00415-023-12148-5

**Published:** 2024-01-09

**Authors:** Augusto J. Mendes, Federica Ribaldi, Aurelien Lathuiliere, Nicholas J. Ashton, Shorena Janelidze, Henrik Zetterberg, Max Scheffler, Frédéric Assal, Valentina Garibotto, Kaj Blennow, Oskar Hansson, Giovanni B. Frisoni

**Affiliations:** 1 Laboratory of Neuroimaging of Aging (LANVIE), University of Geneva, Geneva, Switzerland; 2 Geneva Memory Center, Department of Rehabilitation and Geriatrics, Geneva University Hospitals, Geneva, Switzerland; 3 Department of Psychiatry and Neurochemistry, Institute of Neuroscience and Physiology, the Sahlgrenska Academy at the University of Gothenburg, Mölndal, Sweden; 4 King’s College London, Institute of Psychiatry, Psychology and Neuroscience Maurice Wohl Institute Clinical Neuroscience Institute, London, UK; 5 NIHR Biomedical Research Centre for Mental Health and Biomedical Research Unit for Dementia at South London and Maudsley NHS Foundation, London, UK; 6 Centre for Age-Related Medicine, Stavanger University Hospital, Stavanger, Norway; 7 Clinical Memory Research Unit, Lund University, Lund, Sweden; 8 Clinical Neurochemistry Laboratory, Sahlgrenska University Hospital, Mölndal, Sweden; 9 Department of Neurodegenerative Disease, UCL Institute of Neurology, Queen Square, London, UK; 10 UK Dementia Research Institute at UCL, London, UK; 11 Hong Kong Center for Neurodegenerative Diseases, Clear Water Bay, Hong Kong, China; 12 Wisconsin Alzheimer’s Disease Research Center, University of Wisconsin School of Medicine and Public Health, University of Wisconsin-Madison, Madison, WI USA; 13 Division of Radiology, Geneva University Hospitals, Geneva, Switzerland; 14 Division of Neurology, Department of Clinical Neurosciences, Geneva University Hospitals and Faculty of Medicine, University of Geneva, Geneva, Switzerland; 15 Laboratory of Neuroimaging and Innovative Molecular Tracers (NIMTlab), Geneva University Neurocenter and Faculty of Medicine, University of Geneva, Geneva, Switzerland; 16 Division of Nuclear Medicine and Molecular Imaging, Geneva University Hospitals, Geneva, Switzerland; 17 CIBM Center for Biomedical Imaging, Geneva, Switzerland; 18 Paris Brain Institute, ICM, Pitié-Salpêtrière Hospital, Sorbonne University, Paris, France; 19 Neurodegenerative Disorder Research Center, Division of Life Sciences and Medicine, and Department of Neurology, Institute on Aging and Brain Disorders, University of Science and Technology of China and First Affiliated Hospital of USTC, Hefei, People’s Republic of China; 20 Memory Clinic, Skåne University Hospital, Malmö, Sweden

**Keywords:** Plasma, CSF, *p*-tau, Alzheimer’s disease, Amyloid, Tau

## Abstract

**Background and objective:**

Phosphorylated tau (*p*-tau) 217 has recently received attention because it seems more reliable than other *p*-tau variants for identifying Alzheimer’s disease (AD) pathology. Thus, we aimed to compare the diagnostic accuracy of plasma and CSF *p*-tau217 with *p*-tau181 and *p*-tau231 in a memory clinic cohort.

**Methods:**

The study included 114 participants (CU = 33; MCI = 67; Dementia = 14). The *p*-tau variants were correlated versus continuous measures of amyloid (A) and tau (T)-PET. The *p*-tau phospho-epitopes were assessed through: (i) effect sizes (*δ*) between diagnostic and A ± and T ± groups; (ii) receiver operating characteristic (ROC) analyses in A-PET and T-PET.

**Results:**

The correlations between both plasma and CSF *p*-tau217 with A-PET and T-PET (*r* range 0.64–0.83) were stronger than those of *p*-tau181 (*r* range 0.44–0.79) and *p*-tau231 (*r* range 0.46–0.76). Plasma *p*-tau217 showed significantly higher diagnostic accuracy than *p*-tau181 and *p*-tau231 in (i) differences between diagnostic and biomarker groups (*δ*_range_: *p*-tau217 = 0.55–0.96; *p*-tau181 = 0.51–0.67; *p*-tau231 = 0.53–0.71); (ii) ROC curves to identify A-PET and T-PET positivity (AUC_average_: *p*-tau217 = 0.96; *p*-tau181 = 0.76; *p*-tau231 = 0.79). On the other hand, CSF *p*-tau217 (AUC_average_ = 0.95) did not reveal significant differences in A-PET and T-PET AUC than *p*-tau181 (AUC_average_ = 0.88) and *p*-tau231 (AUC_average_ = 0.89).

**Discussion:**

Plasma *p*-tau217 demonstrated better performance in the identification of AD pathology and clinical phenotypes in comparison with other variants of *p*-tau in a memory clinic cohort. Furthermore, *p*-tau217 had comparable performance in plasma and CSF. Our findings suggest the potential of plasma *p*-tau217 in the diagnosis and screening for AD, which could allow for a decreased use of invasive biomarkers in the future.

**Supplementary Information:**

The online version contains supplementary material available at 10.1007/s00415-023-12148-5.

## Introduction

The main pathological hallmarks of Alzheimer’s disease (AD) are amyloid plaques, neurofibrillary tangles, and neurodegeneration [1]. These biomarkers can be assessed through lumbar puncture to collect and examine cerebrospinal fluid (CSF) or brain imaging (e.g., positron emission tomography [PET] and magnetic resonance imaging [MRI]). Nevertheless, the availability and implementation of these techniques are hampered by practical issues, such as invasiveness and cost. Consequently, a significant number of patients with suspected cognitive impairment caused by AD do not have access to biomarker-supported diagnosis. The use of blood biomarkers, such as plasma phosphorylated tau (*p*-tau), could improve access to AD biomarkers worldwide [2–4]. In fact, this method has the potential to play a crucial role, not only in clinical diagnoses but also in determining eligibility for recently approved therapies.

The dosage of different phospho-epitopes of tau in plasma demonstrated high accuracy in the diagnostic and prognostic evaluation of AD [4, 5]. Plasma *p*-tau181 shows high sensitivity for AD when other neurodegenerative diseases are included in the differential [6, 7]. Additionally, increased *p*-tau181 levels have been observed in early stages of AD [8] and can predict future development of AD dementia [7, 9]. Later on, *p*-tau231 level in plasma was equally found to have a good performance in AD diagnosis, with still earlier increases when compared with *p*-tau181 [10–12].

Most recently, *p*-tau217 has been acclaimed due to the robustness in the identification of AD pathology in comparison with other *p*-tau variants. Plasma *p*-tau217 showed a superior performance to *p*-tau181 when separating AD from other neurogenerative diseases and when detecting AD pathology with either PET or neuropathology [13]. This is of particular interest for an early detection of AD because plasma *p*-tau217 is abnormally elevated in preclinical AD before tau-PET becomes abnormal, and is associated with a steeper cognitive decline [14, 15]. In fact, plasma *p*-tau217 shows the greatest discrimination between amyloid-positive and amyloid-negative cognitively unimpaired (CU) subjects, followed by *p*-tau231 and *p*-tau181 [16]. Moreover, similar findings were observed in a prodromal AD sample, where *p*-tau217 had a higher accuracy in identifying amyloid positivity in patients with mild cognitive impairment (MCI) or those who will progress to AD dementia when compared to the other plasma phospho-epitopes of tau [17].

In line with these findings, CSF *p*-tau217 also revealed superiority compared to CSF *p*-tau181 [18] and *p*-tau231 [19, 20] in the identification of AD pathology. However, the earliest biomarker indicating incipient AD pathology in CSF was *p*-tau231 followed by *p*-tau217 [19]. Likewise, similarly to plasma *p*-tau231, CSF *p*-tau217 also revealed earlier and stronger associations with amyloid and tau derived from PET than plasma *p*-tau181 [10]. Intriguingly, in contrast to other *p*-tau forms that show superiority only in CSF measures to detect amyloid positivity, both plasma and CSF *p*-tau217 exhibit excellent accuracy indices in the diagnostic performance for amyloid pathology [21]. Likewise, the levels of *p*-tau217 found in plasma and CSF also showed similar diagnostic performance in the identification of abnormal levels of tau in PET [13].

Therefore, even if plasma biomarkers are currently thought to represent screening rather than diagnostic biomarkers [22], the ability to measure *p*-tau217 in plasma should rise particular attention as it could find widespread application as a first-line diagnostic and prognostic marker in the near future. In particular, understanding how different phospho-epitopes of tau may have distinct diagnostic validity if detected in plasma or CSF is of special relevance. For that, we intended to compare *p*-tau217 in plasma and CSF to other phospho-epitopes as well as traditional biomarkers in a memory clinic cohort. The current study aimed to (i) evaluate how *p*-tau217 is associated with traditional AD measures of the ATN model (i.e., Centiloid, global tau standardized uptake value ratio [SUVR], and hippocampal volume) and cognition (Mini-Mental State Examination [MMSE] score), (ii) test the diagnostic validity through the differences of plasma and CSF *p*-tau217 in comparison with other *p*-tau variants across different cognitive stages, and (iii) between amyloid and tau positive versus negative individuals; (iv) assess accuracy to detect amyloid and tau PET positivity through receiver operating characteristic (ROC) of plasma and CSF *p*-tau217.

## Methods

### Participants

We selected all participants among patients of the memory clinic of Geneva University Hospitals for whom plasma and/or CSF *p*-tau217, *p*-tau181, and *p*-tau231 were available as well as at least one traditional biomarker (i.e., amyloid-PET, tau-PET, MRI, or CSF), obtained within 18 months. We thus included a total of 114 participants (CU = 33, MCI = 67, dementia = 14; 61% females, mean age = 71.5 [standard deviation (SD) = 7.2]) who had plasma and/or CSF *p*-tau217 measurement and with at least one traditional biomarker available. Nonetheless, the subsample with *p*-tau measurement in CSF was lower, namely of a total of 36 subjects (CU = 10, MCI = 21, dementia = 5). The sample was divided by participants’ cognitive stage, namely, the CU group was composed of all subjects without any cognitive impairment (including worried well, and subjective cognitive decline), while the participants with MCI or dementia were included based on clinical diagnostic criteria and not biomarkers [23, 24]. Every patient underwent diagnostic workup that included clinical and cognitive assessments. Moreover, the evaluation of biomarkers was done based on clinical needs or according to a particular research study a subject was initially enrolled into [25]. Tau-PET was performed on all patients, amyloid-PET was performed in 101 and MRI was obtained in 103 participants. If we consider the subsample with CSF *p*-tau assessment (*n* = 36), all of them underwent tau-PET, 34 amyloid-PET, and 35 underwent MRI. Written informed consent was obtained prior to enrollment in the study from all participants and all procedures were approved by the Geneva Ethics Committee (PB_2016-01346 and 2020_00403).

### Biomarkers collection, analyses, and assessment

#### Plasma and CSF *p*-tau biomarkers

Plasma was collected in EDTA tubes, kept for two hours at room temperature prior to centrifugation (1700 g, 15 min), aliquoted as 500 uL in 1.2 mL polypropylene tubes, and maintained at − 80 °C in the local biobank of the memory clinic of Geneva University Hospitals. Frozen aliquots were sent on dry ice to the Clinical Neurochemistry Laboratory, University of Gothenburg (Sweden), where they were analyzed. The levels of *p*-tau181 [6] and *p*-tau231 [10] were measured using homebrew Simoa assays developed at the Clinical Neurochemistry Laboratory, University of Gothenburg (Sweden). Plasma concentration *p*-tau217 was measured at the Clinical Memory Research Unit, Lund University using Meso Scale Discovery (MSD) based immunoassay developed by Lilly Research Laboratories as previously described [13].

CSF samples was collected at the memory clinic of Geneva University Hospitals following a procedure described elsewhere [25] Likewise, *p*-tau181 and *p*-tau231 measured in CSF were analyzed at Clinical Neurochemistry Laboratory using homebrew Simoa assays, University of Gothenburg (Sweden), whereas *p*-tau217 was processed at the Clinical Memory Research Unit, Lund University using MSD-based immunoassay as previously described [15]. Amyloid and tau positivity were categorized using previously defined CSF cohort-specific thresholds (CSF Aβ42 < 880.5 pg/mL, and CSF *p*-tau181 > 80.5 pg/mL) [26]. CSF Aβ42 (product number 81576) and *p*-tau181 (product number 81574) were analyzed using INNOTEST assays following the manufacturer’s instructions (Fujirebio, Ghent, Belgium) at a chemistry laboratory of Geneva University Hospitals.

#### Neuroimaging biomarkers

Amyloid-PET images were acquired using 18F-florbetapir or 18F-flutemetamol tracers, while tau-PET images were acquired using 18F-flortaucipir using a protocol previously described in detail [27]. In brief, 18F-florbetapir images were acquired 50 min after injection of 200 MBq during 15 min; 18F-flutemetamol images were acquired 90 min after injection of 150 MBq during 20 min; and 18F-flortaucipir images were acquired 75 min after injection of 180 MBq during 30 min. Acquisitions were obtained on Siemens Biograph and Biograph Vision scanners (Siemens, Washington, DC), reconstructed using a 3D OSEM iterative reconstruction, corrected for randoms, dead time, normalization, scatter, attenuation, and sensitivity [27]. An in-house workflow based on SPM12 (Wellcome Department of Cognitive Neurology, London, UK) was used for PET images processing [27]. Considering that we used two different amyloid-PET tracers, SUVR was converted to the Centiloid scale following guidelines from the Global Alzheimer's Association Interactive Network (GAAIN) [28]. The tau-PET global tau SUVR was computed as the average across the parahippocampal gyrus, amygdala, mid-occipital cortex, and inferior temporal cortex [29]. Amyloid-PET (A +) and tau-PET (T +) positivity was assessed visually by an expert in nuclear medicine (VG).

Hippocampal volume was extracted from structural 3T MRI images. The left and right hippocampal volumes were averaged and normalized according to the total intracranial volume. The extraction was performed in FreeSurfer (version 7.0-recon-all; https://surfer.nmr.mgh.harvard.edu).

### Statistical analysis

We evaluated the baseline differences in demographics, clinical, cognitive, and biomarkers among clinical stages using one-way analysis of variance (ANOVA) or Kruskal–Wallis test for non-normally distributed continuous variables, and Fisher’s exact test for categorical variables. In the case of a significant result in the ANOVA (*p* < 0.05), post hoc comparisons were performed using Tukey Honest Significant Differences (HSD), whereas significant results in the Kruskal–Wallis were posteriorly tested using the Dunn test adjusted by Bonferroni technique. The medical records of the participants were examined to assess their cardiovascular health, hypertension, hypercholesterolemia, and diabetes in relation to each *p*-tau variant. The analysis was performed independently to CU and cognitively impaired (CI; composed by MCI and dementia) subjects. Consequently, we conducted a statistical analysis to examine the differences in plasma *p*-tau variants between two subgroups: one with the co-morbidity and the other without. This analysis was performed using the Wilcoxon rank sum test. Furthermore, the correlation between creatinine and *p*-tau levels was examined using Pearson's correlation coefficient.

Likewise, Pearson's correlations were used to assess how each plasma/CSF *p*-tau value correlated with each traditional biomarker (i.e., Centiloid of amyloid-PET, global-SUVR of tau-PET, and hippocampal volume from MRI) and the global cognition score (MMSE).

The diagnostic accuracy of the three *p*-tau variants was assessed through differences for clinical (i.e., CU, MCI, and dementia) and biomarkers groups (i.e., amyloid and tau positivity evaluated through PET: A−/T−, A +/T−, and A +/T +) using the aforementioned non-parametric analysis. In addition to the pairwise comparison using the Dunn test with Bonferroni correction, the non-parametric effect size Cliff's delta (*δ*) was calculated for each significant result in post hoc comparisons.

Additionally, the sensitivity and specificity to identify amyloid and tau positivity for each plasma/CSF *p*-tau variant was estimated. The area under the curve (AUC) of the ROC was calculated for each *p*-tau phospho-epitope and for positivity assessed in PET and CSF. AUC differences between each pair of *p*-tau variants were tested using DeLong’s test. In addition, we calculated the 95% sensitivity, the 95% specificity, and the optimal cut-off derived from the ROC curves to detect amyloid and tau status evaluated in PET. For the optimal cut-off, we used the Youden’s J statistics to maximize the false positive rate (FPR) and the false negative rate (FNR). These analyses were performed using the “*pROC*” package in R [30].

All the analyses were performed in R (R Development Core Team, 2018; R Version 4.2.0).

## Results

### Participants

The mean and SD for the demographic and clinical variables are presented in Table 1. In addition, the test of significance and consequent post hoc comparisons among the three clinical stages revealed a statistically significant decrease in the level of education, MMSE score, and an increase in amyloid-PET positivity and Centiloid, tau-PET positivity and global-SUVR, plasma *p*-tau217, plasma *p*-tau181, and CSF *p*-tau181 (*p* < 0.05) (Table 1). The number of days between plasma or CSF collection and amyloid-PET, tau-PET, MRI, and MMSE is displayed in Table S1 in Supplementary Materials. There were no statistically significant differences observed in the levels of the three plasma *p*-tau variants in CU and CI between the subgroups with and without co-morbidities of cardiovascular disease, hypercholesterolemia, and diabetes (*p* > 0.05; Fig. S1 in Supplementary Materials). On the other hand, *p*-tau217 and *p*-tau231 was significantly decreased in CI participants with hypertension (*p* = 0.02 and *p* = 0.04 respectively), whilst *p*-tau181 did not reveal significant differences in regard to the presence or not of hypertension. Furthermore, we did not find significant correlation between the *p*-tau variants and creatinine levels (*p* > 0.05; Fig. S1 in Supplementary Materials).

**Table 1:** Baseline characteristics of the study cohort by cognitive stage

Demographics	CU (*n* = 33)		MCI (*n* = 67)		Dementia (*n* = 14)		*p*-value
	Mean ± SD	*n*	Mean ± SD	*n*	Mean ± SD	*n*	
Age, years	68.9 ± 7.6	33	72.9 ± 6.4	67	71.1 ± 8.8	14	0.207
Gender, female	21 (64%)	33	33 (49%)	67	7 (50%)	14	0.648
Education, years	16.2 ± 5.1^a^	33	13.9 ± 3.3^b^	67	11.2 ± 3.9^b^	14	< 0.001
MMSE	28.2 ± 1.3^a^	30	26.1 ± 3.1^b^	65	20.1 ± 5.6^c^	14	< 0.001

Imaging	Mean ± SD	*n*	Mean ± SD	*n*	Mean ± SD	*n*	*p*-value
Hippocampal volume	7454 ± 729^a^	30	6993 ± 1022^b^	63	6447 ± 1177^b^	10	0.007
Amyloid PET positivity	6 (21%)^b^	29	41 (66%)^a^	62	9 (90%)^a^	10	< 0.001
Amyloid Centiloid	14.1 ± 34.3^b^	27	48.6 ± 42.2^a^	61	82.2 ± 43.9^a^	10	< 0.001
Tau PET positivity	2 (6%)^b^	33	30 (45%)^a^	67	10 (71%)^a^	14	< 0.002
Tau Global SUVr	1.16 ± 0.17^b^	32	1.35 ± 0.27^a^	66	1.57 ± 0.39^a^	12	< 0.001

Plasma (pg/mL)	Mean ± SD	*n*	Mean ± SD	*n*	Mean ± SD	*n*	*p*-value
*p*-tau217	0.25 ± 0.3^c^	33	0.5 ± 0.42^b^	67	0.7 ± 0.41^a^	14	< 0.001
*p*-tau181	16.3 ± 7.6^b^	26	22.3 ± 10.6	59	25.5 ± 11.1^a^	10	0.018
*p*-tau231	10.7 ± 4.2	26	13.3 ± 6.1	60	12.2 ± 5.2	10	0.427

CSF (pg/mL)	Mean ± SD	*n*	Mean ± SD	*n*	Mean ± SD	*n*	*p*-value
*p*-tau217	13.42 ± 9.96	10	24.29 ± 23.49	21	44.31 ± 31.9	5	0.174
*p*-tau181	313.7 ± 127.8^b^	10	551.9 ± 297.5	20	913.3 ± 624.6^a^	5	0.017
*p*-tau231	700 ± 463	10	1120 ± 1001	20	1549 ± 1065	5	0.309

Table denotes mean ± standard deviation for continuous variables and frequency (percentage) for dichotomous variables

Superscript letters correspond to the results of the post hoc comparison, namely *a* > *b* > *c*

### Correlations between plasma/CSF *p*-tau variants and ATN-C

**Fig. 1:**
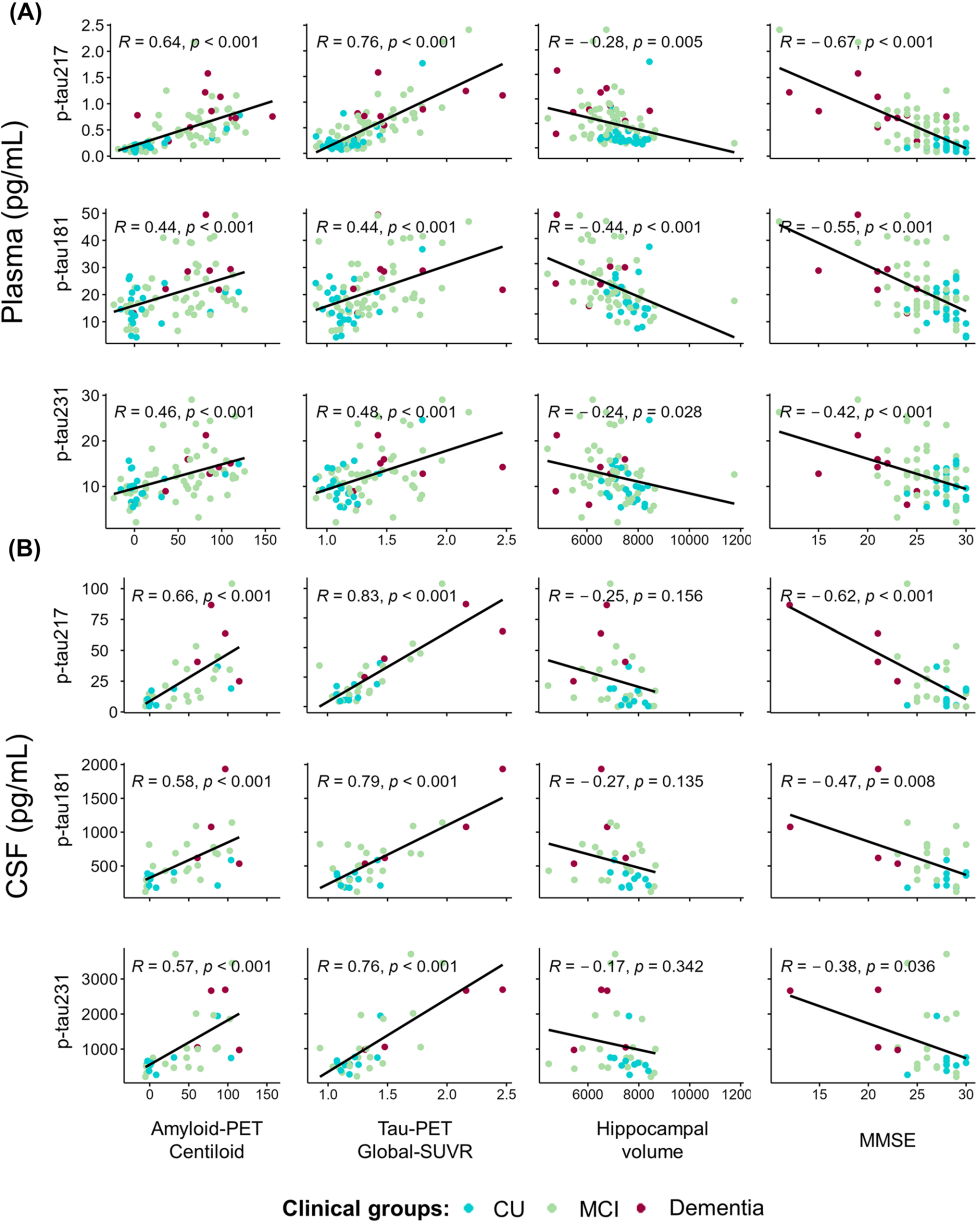
Scatter plots between ATN-C measures and *p*-tau variants measured in plasma and CSF

Pearson correlations revealed significant associations between the three *p*-tau variants measured in plasma and every ATN-C measure (*p* < 0.05). However, the plasma *p*-tau217 showed higher Pearson coefficients in amyloid-PET Centiloid (*r* = 0.64), tau-PET global-SUVR (*r* = 0.76), and MMSE (*r* = − 0.67) than *p*-tau181 (amyloid: *r* = 0.44; tau: *r* = 0.44; cognition: *r* = − 0.55) and *p*-tau213 (amyloid: *r* = 0.46; tau: *r* = 0.48; cognition: *r* = − 0.42) (Fig. 1).

In CSF measures, the three variants of *p*-tau were significant correlated with amyloid, tau and cognition (*p* < 0.05), except for neurodegeneration (*p* > 0.05). In line with plasma results, CSF *p*-tau217 showed higher Pearson correlation with amyloid-PET Centiloid (*r* = 0.66), tau-PET global-SUVR (*r* = 0.83), and MMSE (*r* = − 0.6, *p* < 0.001) in comparison with *p*-tau181 (amyloid: *r* = 0.58; tau: *r* = 0.79; cognition: *r* = − 0.46) and *p*-tau231 (amyloid: *r* = 0.57; tau: *r* = 0.76; cognition: *r* = − 0.37) (Fig. 1).

**Fig. 2:**
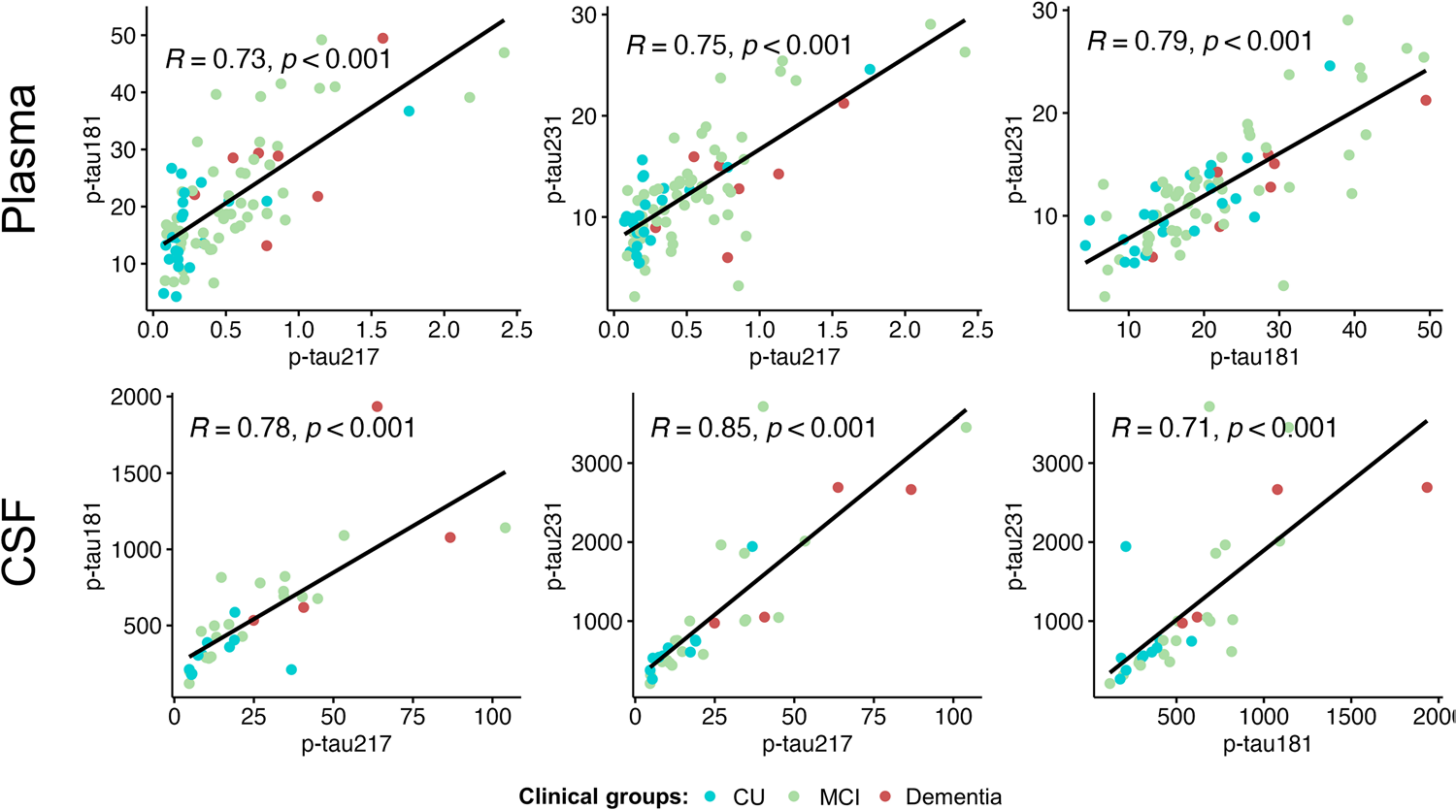
Scatter plots among the different forms of *p*-tau in plasma and CSF

Lastly, the correlations between *p*-tau217 and the other *p*-tau variants were higher when measured in CSF (*p*-tau181: *r* = 0.79, *p* < 0.001; *p*-tau231: *r* = 0.88, *p* < 0.001) in comparison with measures from plasma (*p*-tau181: *r* = 0.67, *p* < 0.001; *p*-tau231: *r* = 0.66, *p* < 0.001). On the other hand, comparisons between *p*-tau181 and *p*-tau231 showed a stronger association for plasma (*r* = 0.79, *p* < 0.001) than for CSF (*r* = 0.72, *p* < 0.001) (Fig. 2).

### Distribution of plasma and CSF *p*-tau levels across cognitive stages

**Fig. 3:**
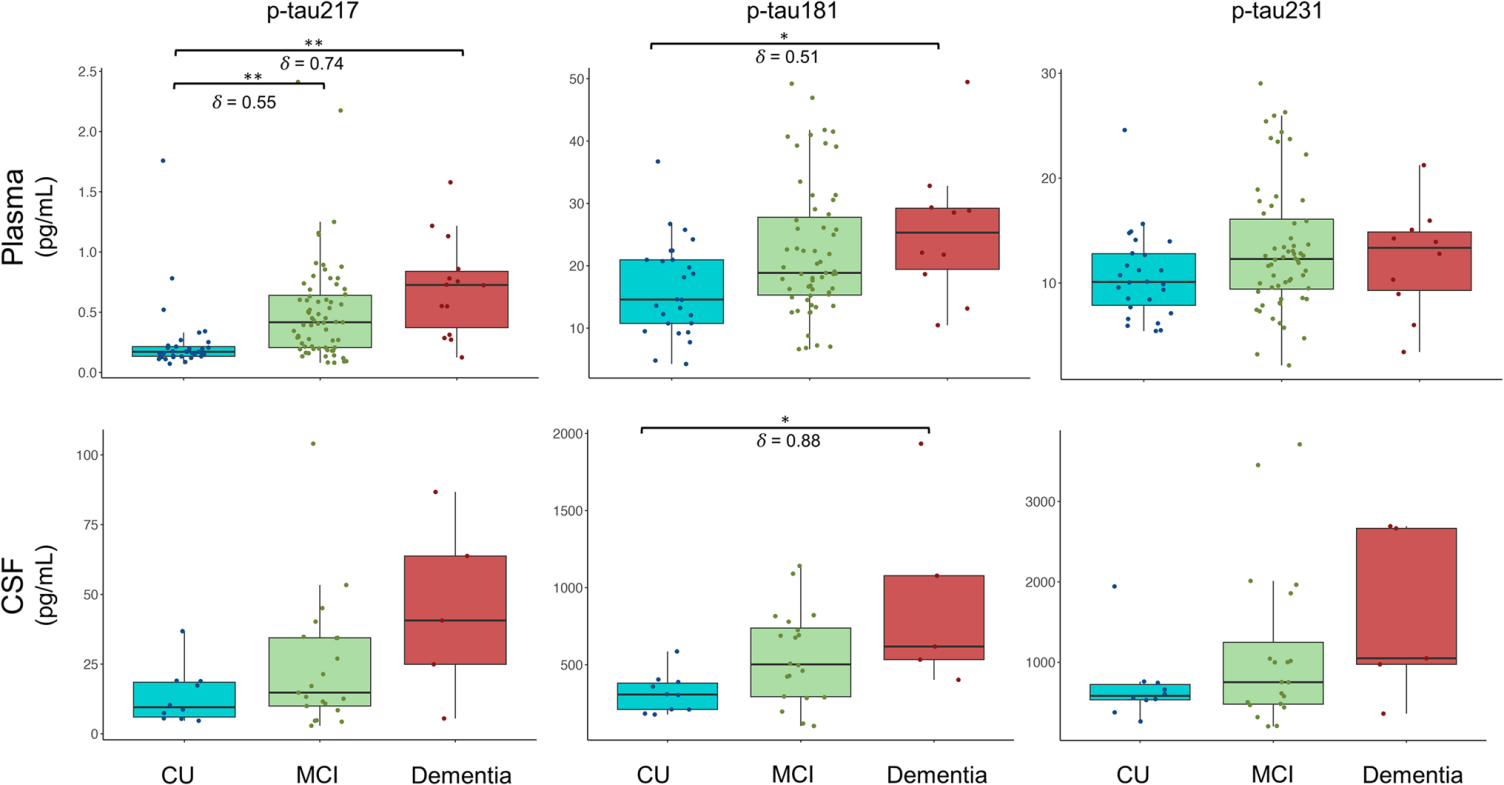
Box plots of plasma and CSF *p*-tau217, *p*-tau181, and *p*-tau231 by cognitive severity. **p < 0.01; *p < 0.05

The Kruskal–Wallis test revealed a statistically significant difference in plasma *p*-tau217 (*χ*^2^(2) = 27.3, *p* < 0.001) and *p*-tau181 (*χ*^2^(2) = 7.9, *p* = 0.018) among the cognitive stages. Post hoc analysis revealed differences in plasma *p*-tau217 between CU and MCI (*p* < 0.001), as well as between CU and dementia (*p* < 0.001). Likewise, differences in plasma *p*-tau181 were observed between CU and dementia (*p* = 0.048) as well a trend towards significance when considering CU and MCI (*p* = 0.052) (Fig. 3). Effect sizes between cognitive stages were higher in plasma *p*-tau217, when compared with *p*-tau181, whereas *p*-tau231 failed to reveal significant differences in this respect (*χ*^2^(2) = 3.5, *p* = 0.17).

For CSF, the Kruskal–Wallis test only showed statistically significant differences in *p*-tau181 (*χ*^2^(2) = 8.1, *p* = 0.018). Post-hoc comparisons revealed significant differences between CU and dementia in CSF *p*-tau181 (*p* = 0.02). Lastly, the Kruskal–Wallis did not reveal significant differences among the three groups for *p*-tau217 (*χ*^2^(2) = 0.12, *p* = 0.11) and *p*-tau231 (*χ*^2^(2) = 3.5, *p* = 0.17) in CSF (Fig. 3).

### Distribution of plasma and CSF *p*-tau levels according to amyloid and tau positivity

**Fig. 4:**
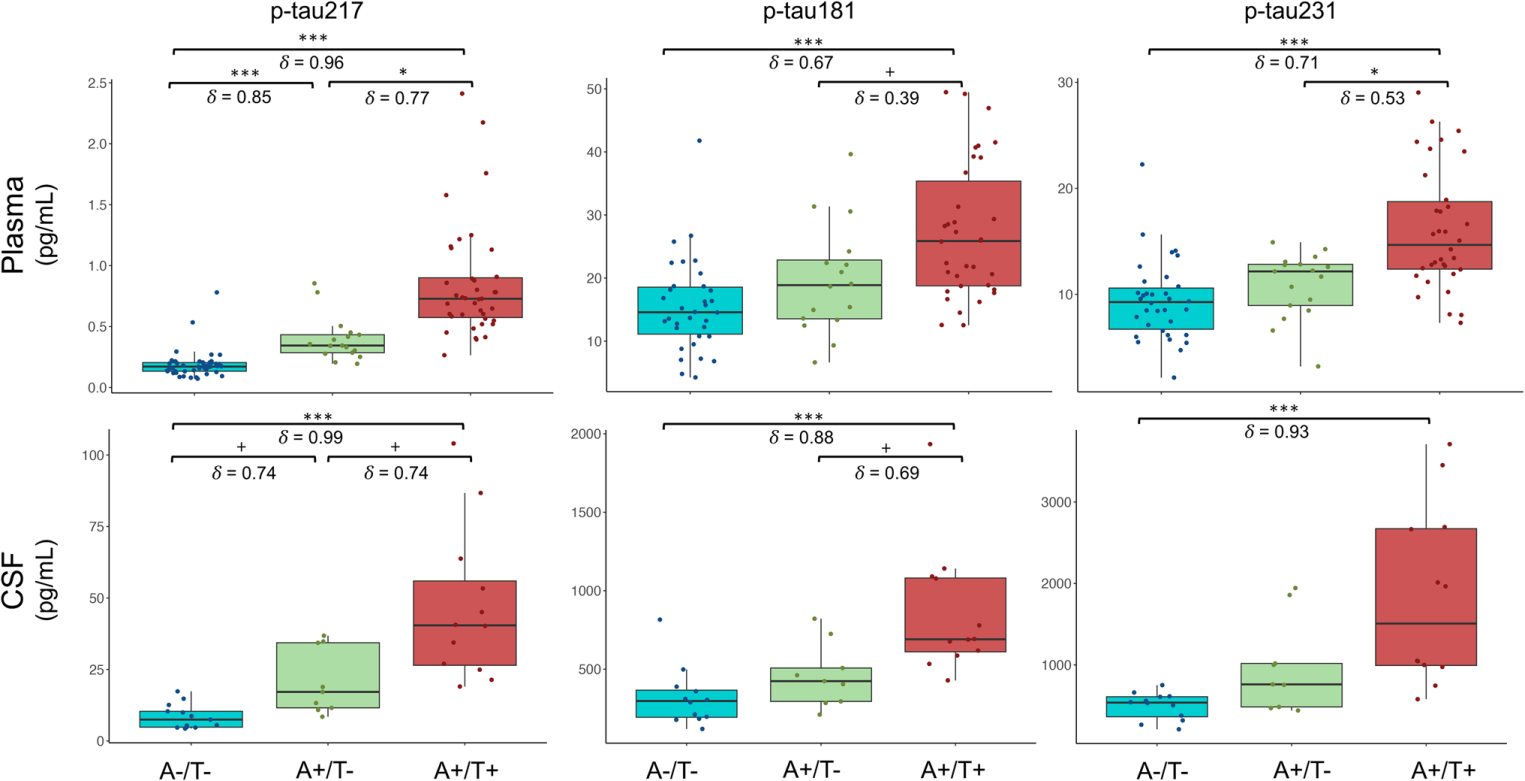
Box plots of plasma and CSF *p*-tau217, *p*-tau181, and *p*-tau231 according to the positivity in amyloid and tau-PET. ***p < 0.001; **p < 0.01; *p < 0.05; +  < 0.1

The three biomarker groups from our cohort comprised 54 A−/T−, 17 A +/T−, and 39 A +/T + subjects with plasma *p*-tau217 measurements. There was a statistically significant difference in plasma *p*-tau217 (*χ*^2^(2) = 71.9, *p* < 0.001), *p*-tau181 (*χ*^2^(2) = 22.9, *p* < 0.001), and *p*-tau231 (*χ*^2^(2) = 28.3, *p* < 0.001) among biomarker groups. Post-hoc analysis revealed differences between A +/T + and A−/T− for the three plasma *p*-tau variants (*p* < 0.001) as well as significant statistically differences between A +/T + and A +/T− in *p*-tau217 (*p* = 0.02) and *p*-tau231 (*p* = 0.01). Furthermore, only plasma *p*-tau217 showed a significant difference between A−/T− and A +/T− subjects (*p* < 0.001). The effect size *δ* revealed higher standardized differences among biomarker groups in plasma *p*-tau217 (*δ* from 0.77 to 0.96), when compared with *p*-tau181 (*δ* from 0.39 to 0.67), and with *p*-tau231 (*δ* from 0.53 to 0.71) (Fig. 4).

On the other hand, the number of subjects with CSF *p*-tau217 measurement was lower, namely 12 A−/T−, 9 A +/T−, and 12 A +/T + subjects. Similarly, Kruskal–Wallis analysis revealed significant differences in CSF *p*-tau217 (*χ*^2^(2) = 23.2, *p* < 0.001), *p*-tau181 (*χ*^2^(2) = 15.9, *p* < 0.001), and *p*-tau231 (*χ*^2^(2) = 16.7, *p* < 0.001) among biomarker groups. The post-hoc analysis showed significant differences in all *p*-tau variants between A +/T + and A−/T− (*p* < 0.001), whereas a trend towards significance was observed in *p*-tau217 and *p*-tau181 between A +/T + and A +/T− (*p* < 0.1). At last, a trend towards significance was also observed between A−/T− and A +/T− subjects in *p*-tau217 (*p* = 0.07), while no significant differences were observed in the other variants (*p* > 0.05). Contrarily to plasma, the effect size *δ* was comparable between CSF *p*-tau217 (*δ* from 0.74 to 0.99), *p*-tau181 (*δ* from 0.69 to 0.88), and *p*-tau231 (*δ* = 0.88) (Fig. 4).

### Sensitivity and specificity of plasma/CSF *p*-tau217 in amyloid/tau positivity

**Fig. 5:**
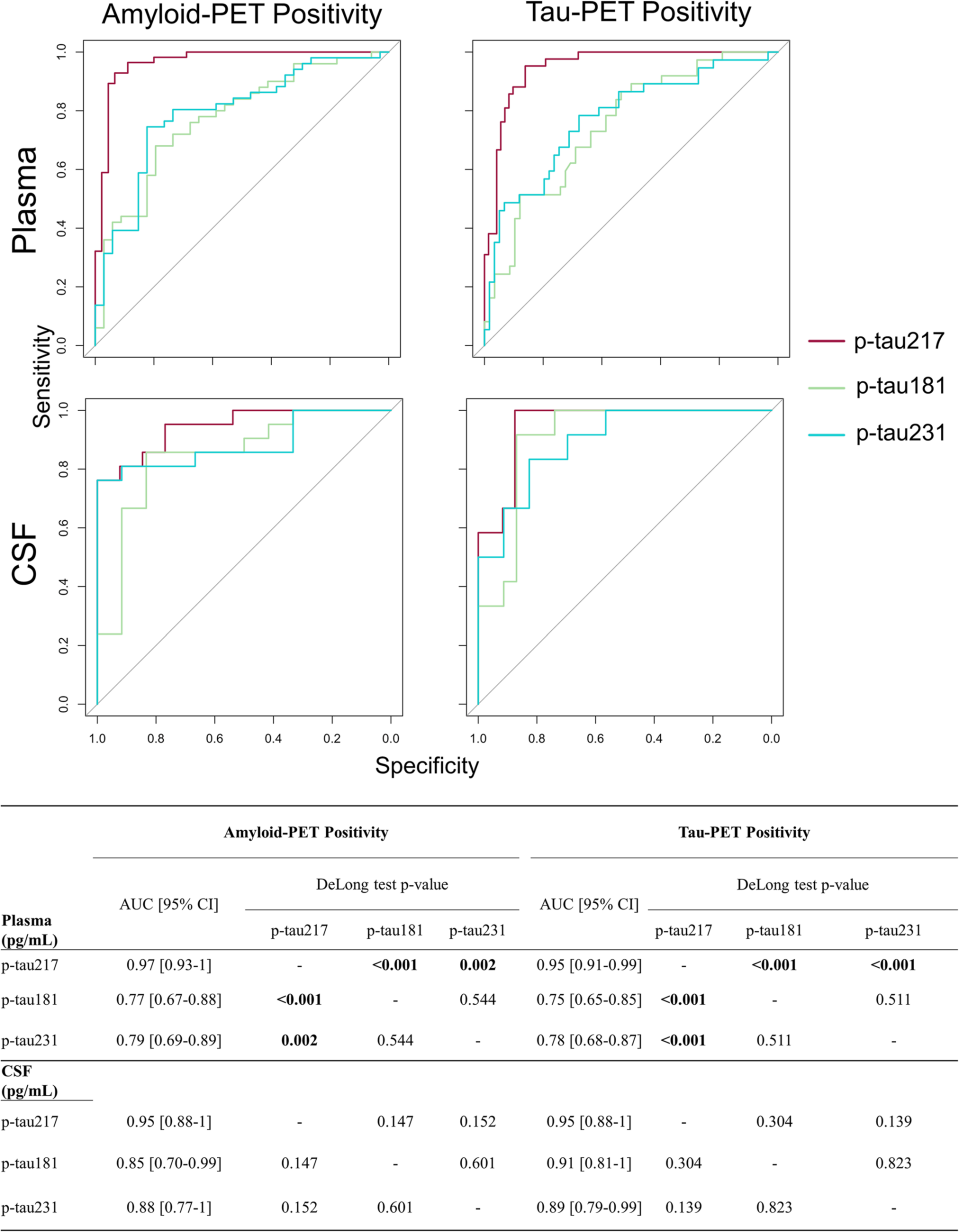
Classification accuracy of the three variants of *p*-tau measured in plasma and CSF in amyloid and tau positivity evaluated in PET

The accuracy to detect the positivity from amyloid-PET was higher in plasma *p*-tau217 in comparison with *p*-tau181 (*p* < 0.001) and *p*-tau231 (*p* = 0.002). Likewise, the accuracy in classifying tau positivity from tau-PET was superior in plasma *p*-tau217 than in *p*-tau181 and *p*-tau231 (*p* < 0.001). However, CSF *p*-tau217 did not revealed significant differences with *p*-tau181 or *p*-tau231 in the identification of positive subjects in amyloid-PET and tau-PET positivity (*p* > 0.05) (Fig. 5). On the other hand, we did no find significant differences among the three *p*-tau variants measured in plasma and CSF in the detection of the positivity of amyloid (*p* > 0.05) and tau (*p* > 0.05) measured in CSF (Fig. S2 in Supplementary Materials).

**Fig. 6:**
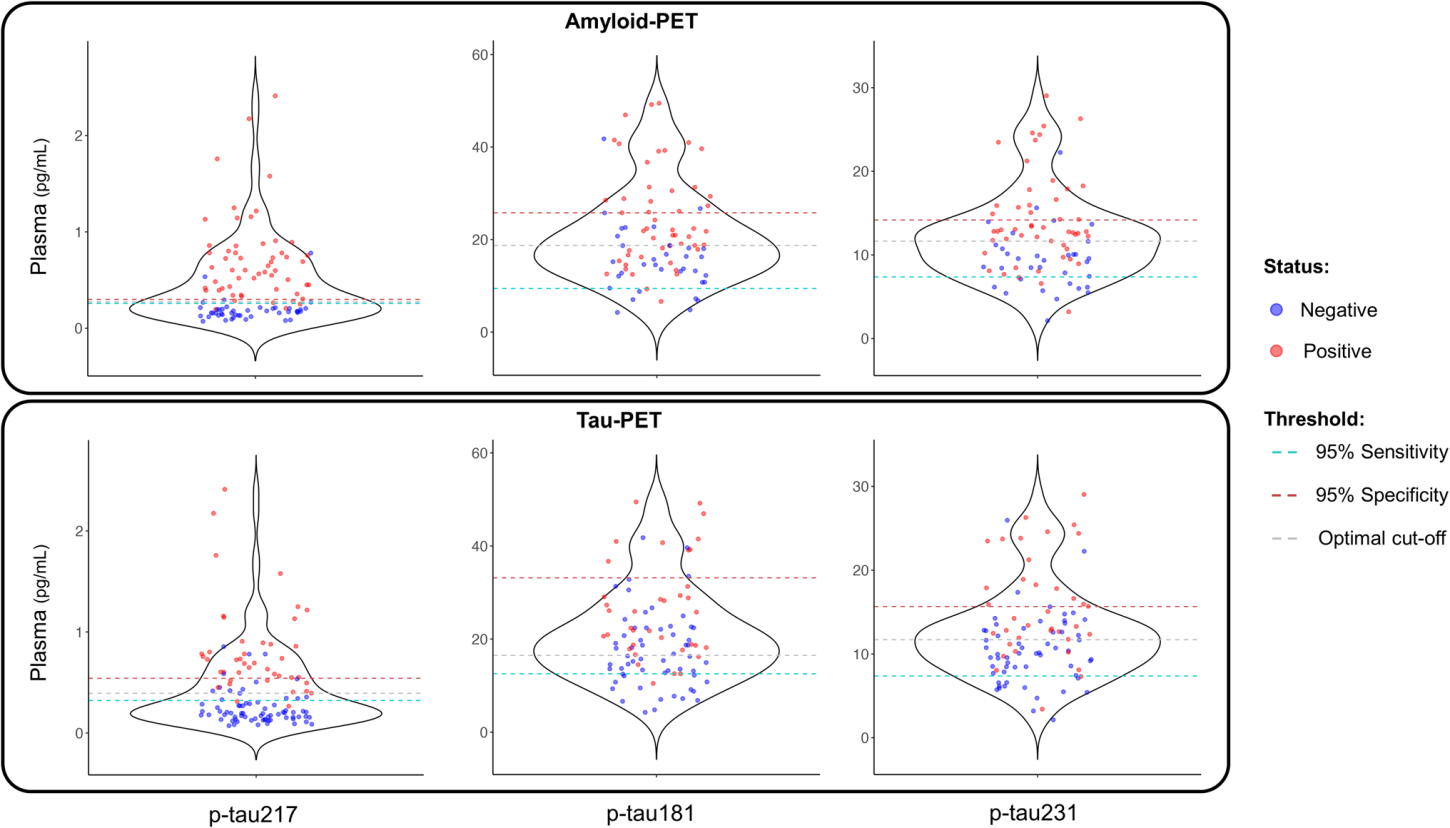
Violin plots for the three *p*-tau variants evaluated in plasma with the 95% sensitivity, 95% specificity, and optimal cut-off over the amyloid and tau status assessed in PET

Lastly, we have shown that the implementation of a cut-off for plasma *p*-tau217 of 0.27 pg/mL within our study cohort for amyloid-PET positivity yields a FPR of 6.7% and a FNR of 7.1%. The optimal threshold for plasma *p*-tau217 in determining tau-PET positivity was found to be 0.38 pg/mL, with a FPR of 23.9% and a FNR of 4.7% (Fig. 6). The other plasma *p*-tau variants cut-offs demonstrated higher FPR and FNR in the identification of positive results for both amyloid and tau-PET (Fig. 6 and Table S2 in Supplementary Materials). The best cut-offs for the three variants of *p*-tau measured in CSF revealed comparable FPR and FNR. For instance, to identify amyloid positivity, the three variants demonstrated a FPR that ranged from 0 to 16.7% and a FNR between 14.3 and 23.8% (Fig. S3 in Supplementary Materials). Conversely, the utilization of CSF *p*-tau217 demonstrated superior sensitivity and specificity in the detection of tau-PET positivity (FPR = 12.5%; FNR = 0%), followed by *p*-tau181 (FPR = 13.1%; FNR = 8.3%), and *p*-tau231 (FPR = 17.4%; FNR = 16.7%).

## Discussion

The current study showed that plasma *p*-tau217 is more efficient than *p*-tau181 and *p*-tau231 in identifying amyloid and tau positivity, as assessed by PET imaging, in a group of participants from a memory clinic cohort. In fact, plasma *p*-tau217, in comparison with other epitopes of *p*-tau, showed larger associations with Centiloid values derived from amyloid-PET, global tau SUVR measured in tau-PET, and global cognition assessed with the MMSE. On the other hand, *p*-tau217 measured in CSF also revealed higher correlations with Centiloid, global-SUVR, and MMSE, even though the superiority in diagnostic performance was less compelling. Furthermore, we have noticed larger differences in plasma and CSF *p*-tau217 levels throughout different cognitive stages and within groups classified as A +/T + positive. These findings are consistent with our ROC analysis highlighting the high sensitivity and specificity of *p*-tau217 in identifying individuals who exhibit amyloid and tau positivity. Overall, our results suggest *p*-tau217 is an appropriate biomarker for monitoring the presence of amyloid and tau, as it is particularly low in subjects without AD pathology and increases rapidly over the significance threshold with the occurrence of amyloidosis and tau pathology.

In particular, *p*-tau217 measured in peripheral blood revealed high correlations with traditional AD biomarkers and elevated accuracy in the identification of amyloid and tau in PET (AUC > 90). This is consistent with a recent longitudinal study that observed an increase in plasma *p*-tau217 over time for amyloid-positive subjects, whereas it remained stable in amyloid-negative subjects [31]. Additionally, when *p*-tau217 was compared to several other plasma biomarkers (such as *p*-tau231, *p*-tau181, amyloid-β 42/40, glial acidic fibrillary protein, and neurofilament light), it was found to be the only biomarker with longitudinal changes dependent on amyloid as well as associations with cognitive impairment and neurodegeneration [11]. Likewise, when compared with tau-PET and plasma *p*-tau181, *p*-tau217 levels were the best predictors of tau-PET accumulation in amyloid-positive CU subjects [32]. Thus, our findings confirm previous studies suggesting that plasma *p*-tau217 is more effective in detecting AD pathology than *p*-tau181 [13, 16, 33–35] and *p*-tau231 [17, 21].

On the other hand, CSF *p*-tau217 also revealed higher AUC in the detection of AD pathology in comparison with other CSF *p*-tau variants, even though the test of significance did not detect any statistically significant difference. However, we should acknowledge a potential constraint of statistical power given that the CSF subsample was smaller than the total sample (see “Limitations” section). In fact, our descriptive results are in line with previous studies suggesting higher performance of CSF *p*-tau217 in comparison with CSF *p*-tau181 [18] and *p*-tau231 [19, 20]. This is of particular interest because, even though we observed significant correlations among the three *p*-tau variants measured in CSF, low correlations between plasma *p*-tau217 and other variants of *p*-tau were observed, whereas plasma *p*-tau181 and *p*-tau231 demonstrated a high correlation coefficient. These findings indicate a distinction between *p*-tau217 and both other variants when quantified in plasma, however, the differentiation is less pronounced when evaluated in CSF. The disparity in plasma and CSF *p*-tau variants along the disease continuum could account for this phenomenon [36–38], despite the fact that all of the tested *p*-tau variants have been associated with amyloid changes in preclinical AD [38, 39].

Moreover, plasma *p*-tau217 and the three *p*-tau variants measures in CSF revealed higher correlation coefficient with global tau SUVR than with amyloid-PET Centiloid. For the three CSF *p*-tau variants, a recent study has shown stronger associations with amyloid-PET instead of tau-PET SUVR, while the microtubule-binding region (MTBR) tau243 was the variant more strongly associated with tau phosphorylation [40]. However, in line with our findings, CSF *p*-tau217 was higher correlated with amyloid- and tau-PET when compared with *p*-tau181 and *p*-tau231 [20, 40]. Likewise, plasma *p*-tau217 also appears to be a marker of more of amyloid rather than of tau pathology [41]. Nonetheless, the dynamics change along the AD continuum. If plasma *p*-tau217 has already shown to be strongly associated with amyloid-PET in the early stages of AD, stronger correlations with tau-PET are observed in later stages [42]. Taken together, these findings also suggest the similarity between plasma and CSF *p*-tau217 in contrast to the disparities shown for *p*-tau181 and *p*-tau231 when measured in plasma versus CSF.

In fact, CSF and plasma *p*-tau217 have already shown equivalent performance in the identification of AD pathology [13, 21], while *p*-tau181 and *p*-tau231 measured in CSF had a higher AUC in identifying amyloid and tau in PET, in comparison to plasma measurements [38, 39]. The advantage of CSF over plasma could be due to the fact that CSF surrounds the brain parenchyma directly and functions as a drainage system for brain extracellular solutes [43]. Nonetheless, our findings indicate a comparable performance between plasma and CSF *p*-tau217 which highlights the potential of the plasma biomarker to improve accessibility to an accurate diagnostic tool, and to decrease the invasive character of the disease’s workup.

### Limitations

One of the main limitations is the small sample size with CSF collection (*n* = 36), which makes it difficult to properly compare the plasma findings with CSF. Moreover, the CSF cut-offs applied to define positivity were based on cohort-specific previous measures [26]. However, the specificity and sensitivity results were very similar to the ones derived using amyloid and tau-PET, which suggests the feasibility of the proposed cut-offs for our cohort. Likewise, it is important to acknowledge that each participant did not undergo an identical diagnostic protocol. For instance, even though all participants performed tau-PET in temporal proximity to plasma and CSF collection, only 101 out of 114 participants underwent an amyloid-PET scan (see Table 1).

Additionally, as would be expected in a cohort from a memory clinic, the majority of the patients in our sample had MCI, which creates an imbalance of the number of subjects in each cognitive stage. Moreover, our CU group also presented a higher proportion of amyloid positive subjects (i.e., 21%), which is a typical characteristic of memory clinic cohorts [44]. As a result, even if it would be challenging to apply our findings to a larger community, they could prove to be very helpful when applied to a different memory clinic population, which is mainly composed of MCI and/or amyloid-positive patients.

### Conclusion

The current study suggests *p*-tau217 as a suitable biomarker for identifying AD pathology. Compared to other variants of *p*-tau, the levels of *p*-tau217 in plasma were better at identifying amyloid and tau-PET positivity. Particularly, *p*-tau217 measured in plasma and CSF showed similar AUCs in the classification of amyloid and tau positivity, whereas *p*-tau181 and *p*-tau231 assessed in CSF revealed higher performance in comparison with their plasma correspondents. Moreover, our findings showed that plasma and CSF *p*-tau217 had stronger associations with amyloid, tau and neurodegeneration, and cognition when compared to *p*-tau181 and *p*-tau231. Our results for CSF data were less compelling, but this could possibly be explained by the limited number of analyzed subjects, when compared to plasma. Future studies should enroll more evenly balanced samples when realizing a comparison between both techniques.

### Supplementary Information

Below is the link to the electronic supplementary material.

Supplementary file 1 (PDF 630 KB)
