# Supplementary material for: Head-to-head study of diagnostic accuracy of plasma and cerebrospinal fluid p-tau217 versus p-tau181 and p-tau231 in a memory clinic cohort
Source: J Neurol. 2024 Jan 9;271(4):2053–66. doi: 10.1007/s00415-023-12148-5 (PMC10972950; doi:10.1007/s00415-023-12148-5)
Supplement: Supplementary file 1 — (PDF 630 KB) [file 415_2023_12148_MOESM1_ESM.pdf]

Supplementary Materials

Head-to-head study of diagnostic accuracy of plasma and cerebrospinal fluid p-tau217 versus p-tau181 and p-tau231 in a memory clinic cohort

Augusto J. Mendes, PhD; Federica Ribaldi, PhD, Aurelien Lathuiliere, MD, PhD; Nicholas J. Ashton, PhD; Shorena Janelidze, PhD; Henrik Zetterberg, MD, PhD; Max Scheffler, MD; Frédéric Assal, MD; Valentina Garibotto, MD; Kaj Blennow, MD; Oskar Hansson, MD, PhD; Giovanni B. Frisoni, MD

CONTENT

Figure S1. Boxplots for each plasma p-tau variant in CI and CU according to their cardiovascular, hypercholesterolemia, hypertension, and diabetes history (A-D) and scatter plot between each p-tau concentration and creatinine levels (E).

Table S1. Number of days between the collection of plasma or CSF and traditional biomarkers plus MMSE (Median ± Interquartile Range [IQR]).

Figure S2. Classification accuracy of the three isoforms of p-tau measured in plasma and CSF in amyloid and tau positivity evaluated in CSF.

Table S2. The optimal cut-off using Youden's J for each p-tau variant and underlying sensitivity, specificity, FPR, FNR, and accuracy according to amyloid and tau positivity derived from PET.

Figure S3. Violin plots for the three p-tau variants evaluated in CSF with the 95% sensitivity, 95% specificity, and optimal cut-off over the amyloid and tau status assessed in PET.

Supplementary Materials

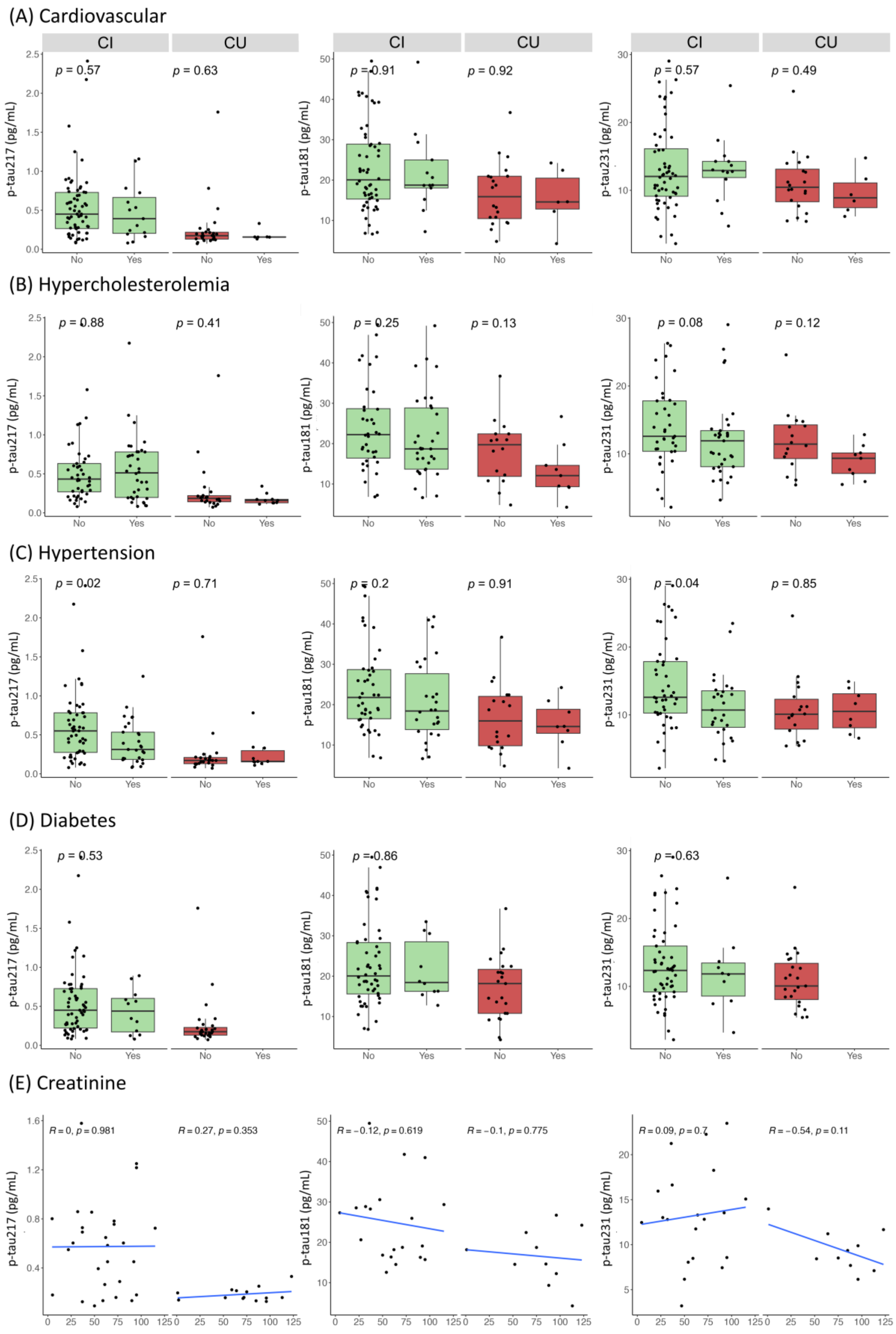

Figure S1. Boxplots for each plasma p-tau variant in CI and CU according to their cardiovascular, hypercholesterolemia, hypertension, and diabetes history (A-D) and scatter plot between each p-tau concentration and creatinine levels (E).

Table S1. Number of days between the collection of plasma or CSF and traditional biomarkers plus MMSE (Median ± Interquartile Range [IQR]).

	Plasma	CSF
Amyloid-PET	95 ± 199	32 ± 56
Tau-PET	97 ± 153	101 ± 207
MRI	26 ± 91	33 ± 67
MMSE	73 ± 118	50 ± 63
Plasma	-	26 ± 91
CSF	26 ± 91	-

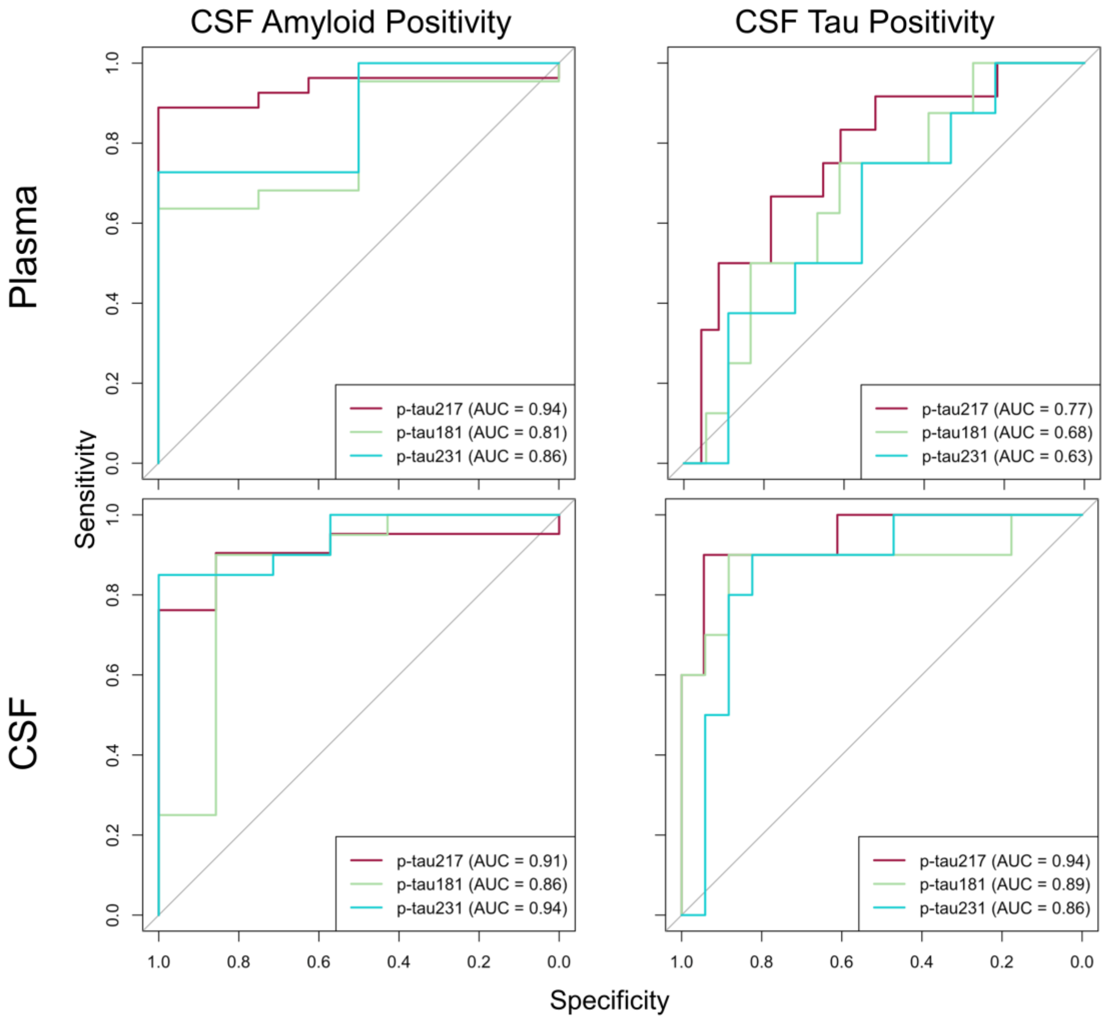

Figure S2. Classification accuracy of the three isoforms of p-tau measured in plasma and CSF in amyloid and tau positivity evaluated in CSF.

Table S2. The optimal cut-off using Youden's J for each p-tau variant and underlying sensitivity, specificity, FPR, FNR, and accuracy according to amyloid and tau positivity derived from PET.

	Amyloid-PET Positivity						Tau-PET Positivity					
Plasma (pg/mL)	Cut-off	Sensitivity	Specificity	FPR	FNR	Accuracy	Cut-off	Sensitivity	Specificity	FPR	FNR	Accuracy
p-tau217	0.27	92.9	93.3	6.7	7.1	93.1	0.39	95.2	86.1	23.9	4.7	89.5
p-tau181	18.73	68	79.4	20.6	32	72.6	16.5	86.5	53.4	46.6	13.5	66.3
p-tau231	11.65	74.5	82.4	17.6	25.5	77.6	11.7	78.4	67.8	32.2	21.6	71.9
CSF (pg/mL)												
p-tau217	18.12	76.2	100	0	23.8	85.3	18.9	100	87.5	12.5	0	91.7
p-tau181	396.5	85.7	83.3	16.7	14.3	84.8	520.5	91.7	86.9	13.1	8.3	88.6
p-tau231	753.3	76.2	100	0	23.8	84.8	868.2	83.3	82.6	17.4	16.7	82.9

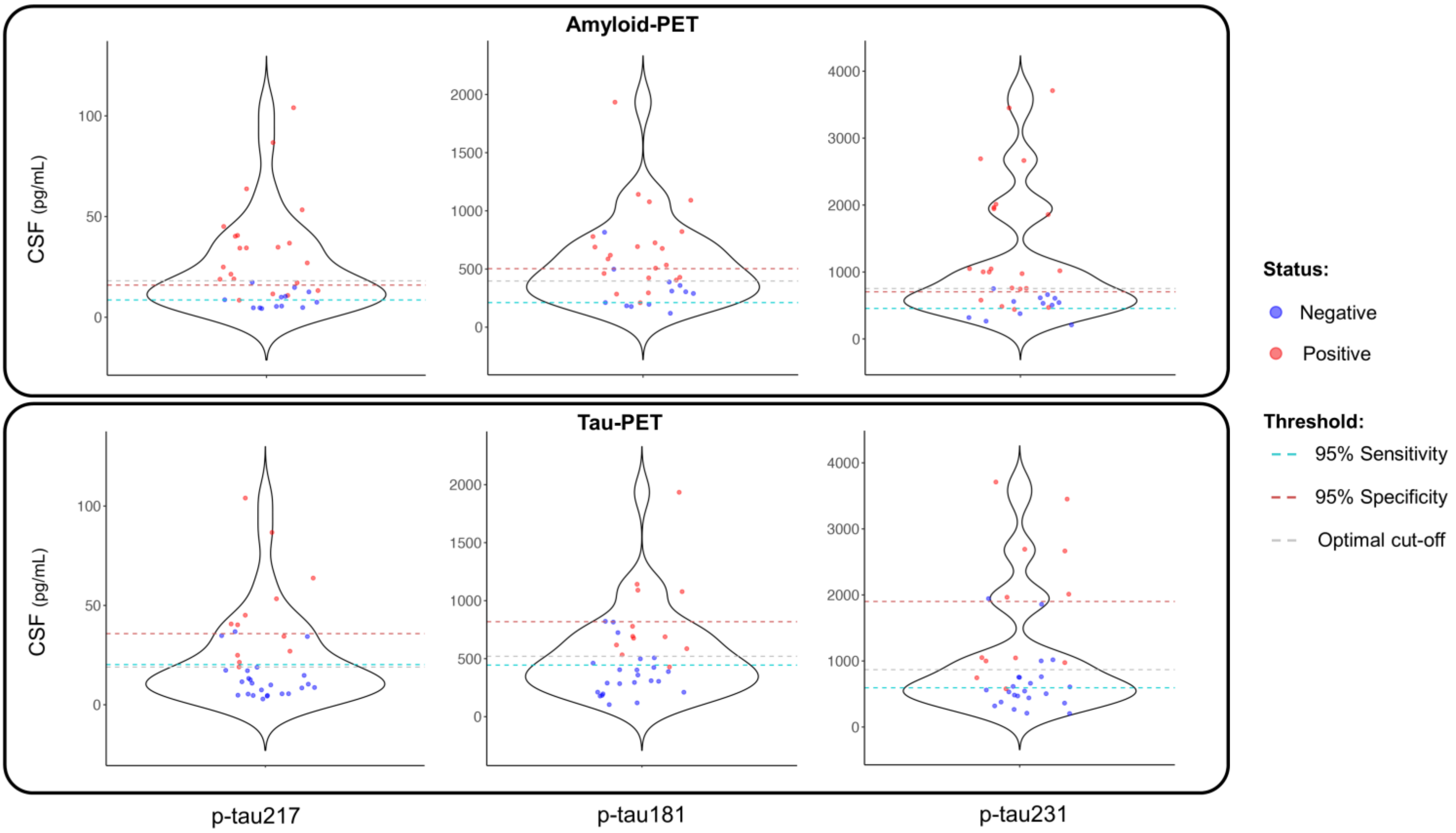

Figure S3. Violin plots for the three p-tau variants evaluated in CSF with the 95% sensitivity, 95% specificity, and optimal cut-off over the amyloid and tau status assessed in PET.
